# Nano-Enhanced Polymer Composite Materials: A Review of Current Advancements and Challenges

**DOI:** 10.3390/polym17070893

**Published:** 2025-03-26

**Authors:** Abdulrahman Adeiza Musa, Abdulhakeem Bello, Sani Mohammed Adams, Azikiwe Peter Onwualu, Vitalis Chioh Anye, Kamilu Adeyemi Bello, Ifeyinwa Ijeoma Obianyo

**Affiliations:** 1 Department of Metallurgical and Materials Engineering, Ahmadu Bello University, Zaria 810107, Nigeria; 2 Department of Materials Science and Engineering, African University of Science and Technology, Abuja 900107, Nigeria; abello@aust.edu.ng (A.B.); aonwualu@aust.edu.ng (A.P.O.); vanye@aust.edu.ng (V.C.A.); 3 Department of Metallurgical and Materials Engineering, University of Nigeria, Nsukka 410105, Nigeria; 4 Department of Civil Engineering, Nile University of Nigeria, Abuja 900108, Nigeria

**Keywords:** nanocomposite, carbon-based nanomaterials, composite development, challenges, performance enhancement

## Abstract

Nanomaterials have demonstrated significant potential in enhancing the performance and functionality of composite materials across various industrial applications. This review delves into the unique properties of nanomaterials, with a particular focus on carbon-based nanomaterials, and presents key findings on their effectiveness in improving composite performance. The study emphasizes specific nano-based composite materials, highlighting their substantial promise in advancing the field of nanocomposites. Additionally, it addresses the challenges associated with the production and utilization of nanocomposite materials and discusses potential solutions to overcome these obstacles. The review concludes with recommendations for further research and innovation in nanocomposites to fully harness the advantages of these advanced materials for broader future applications.

## 1. Introduction

Composite materials play an important role in addressing the evolving needs of various industries, ranging from aerospace and automotive to construction and electronics. They offer a unique combination of properties, such as a high strength-to-weight ratio, excellent corrosion resistance, good thermal stability, and remarkable design flexibility, making them suitable for diverse applications in critical sectors, including sports, marine, and biomedicine [1,2,3]. The pursuit of ever-improving material performance has led researchers to explore novel avenues to further enhance the performance of composite materials to meet contemporary sectoral demands.

Recent advances in nanotechnology have paved the way for the development of functional composite materials with enhanced product efficiency, cost-effectiveness, and overall performance improvement [4]. Properties such as thermal stability, electrical conductivity, strength, and stiffness-to-weight ratio have been significantly improved by introducing different nanomaterials into composite structures [5]. Nanocomposites, composite materials reinforced with nanoparticles or nanofillers, have the potential to revolutionize many advanced engineering sectors due to their unique surface properties, unprecedented levels of functionality, and exceptional performance. Nano-based materials, such as carbon nanotubes (CNTs), graphene, fullerenes, and nanocellulose, possess distinctive characteristics that have captured the interest of researchers. They have harnessed the potential of these materials to realize significant advantages across various sectors, including composite design and development. This strategy entails the optimal integration of nano-based materials into composites to meet specific performance criteria and address the escalating demand for increasingly complex materials across diverse sectors. Industries such as aerospace, automotive, marine, and biomedicine require high-performance composite materials for manufacturing components that require multifunctionality [6,7]. The emergence of nanocomposite materials is progressively addressing these demands thanks to their unique combination of properties, in addition to their lightweight nature.

Effective interfacial interactions among individual constituents are a critical factor in determining the overall performance of composite materials [8]. Nano-based materials have the potential to enhance interfacial bonding between the matrix and reinforcement during composite fabrication. This is attributed to the distinctive surface properties of these materials at the nanoscale level [9]. The exceedingly small size of nano-based materials, coupled with their high surface area and energy, enhances their physical and chemical activities. This characteristic enables them to bond more readily with other surfaces than materials at the macro or micro levels [10]. Nanomaterials have been widely used to improve the properties of composite materials. The effectiveness of these nanomaterials in enhancing the specific characteristics of polymer-based composites depends on the targeted application area [11,12]. Ma et al. [13] employed an analytical model to evaluate the effectiveness of interfacial interactions between multiphase carbon nanotubes and thermoplastic resin in developing high-performance nanocomposites for multifunctional applications.

Nanomaterials exhibit attractive characteristics suitable for influencing a broad spectrum of properties in nanocomposite development [14]. The integration of nanomaterials into composites has led to improvements in mechanical strength, durability, electrical, thermal, and optical performances, paving the way for their high demand in critical applications such as engineering, transportation, biomedical, and pharmaceutical sectors [15,16,17,18]. In recent years, the engineering sector has witnessed tremendous growth in the development of robust materials for lightweight structural applications, including aerospace, automotive, and construction industries, due to the novel characteristics of nanocomposite materials.

In addition to the lightweight advantages of nanomaterial-based composites, their excellent electrical conductivity and thermal stability significantly improve the efficiency and safety of electronic components and thermal management systems in vehicles and aircraft [19,20]. The use of nanomaterials in the biomedical and pharmaceutical sectors has been extensively explored, as widely reported in the literature [21,22,23,24]. Nanocomposites offer unique opportunities for the development of advanced materials with improved biocompatibility and tailored functionalities, leading to more effective treatment methods with fewer side effects. This includes novel biocompatible implants, drug delivery systems, and diagnostic tools [25,26]. The unique properties of nanomaterials have been attributed to their high surface-area-to-volume ratio, tunable surface chemistry, enhanced reactivity, and quantum confinement effects [27,28]. Nanomaterials are manufactured at the nanoscale, having at least one dimension less than 100 nm. Recently, carbon-based nanomaterials have attracted significant attention as nanofillers for developing high-performance composites in many applications [29,30,31,32,33]. Composite materials in which at least one of the components has a dimension in the nanoscale range are referred to as nanocomposites. Nanocomposites are typically composed of a polymer, ceramic, or metal matrix reinforced with nano-based materials such as nanoparticles or nanofibers. Reports have shown that the advent of polymer-based nanocomposites has greatly revolutionized many critical sectors, including water treatment, food processing, transportation, and biomedical science, due to their lightweight advantage compared to metal and ceramic counterparts [34,35]. These include both petroleum-derived polymers and bioplastics.

However, the difficulty in controlling the dispersion and alignment of nanomaterials during composite production has remained a significant challenge for researchers and manufacturers [36,37]. Moreover, concerns about their potential environmental impact further complicate the exploration and use of nanomaterials in the production of composites for functional applications.

In this study, we provide a summary of recent advances in nanocomposite development and their potential for advanced applications. First, some emerging nanomaterials and their attractive characteristics in terms of advancing composite performance are highlighted. Subsequently, research progress on using various nano-based materials to enhance the mechanical, electrical, thermal, and optical performance of nanocomposites is presented. This study further explores the challenges posed by nanomaterials during composite production and their potential environmental impact during and after end-of-life use. Finally, we offer suggestions for future research directions to address the current gaps in research by developing novel and eco-friendly nano-based composites and adopting more sustainable practices in the composite manufacturing sector. 

### 1.1. Nanomaterials

Nanomaterials refer to materials with a size of approximately 1–100 nm and dimensions in the nanometer range [38]. Nanomaterials are among the most advanced materials in recent technological development [39]. The numerous functions and special physicochemical characteristics of nanomaterials make them useful for sustainable technology. In general, nanomaterials can be classified into three major groups: organic-based nanomaterials, inorganic-based nanomaterials, and carbon-based nanomaterials [40]. Several nanomaterials have been successfully used to improve the properties of composite materials for advanced applications, including carbon nanotubes, graphene, nanocellulose, metal nanoparticles, ceramic nanoparticles, and polymer-based nanoparticles [41,42,43,44].

#### 1.1.1. Organic Nanomaterials

Organic-based nanomaterials are a class of materials made of organic compounds or containing organic components that combine the unique properties of nanoscale structures with the flexibility and functionality of organic molecules [39,45]. These materials can be synthesized from various organic compounds, including polymers, lipids, peptides, and nucleic acids, using different techniques such as chemical vapor deposition (CVD), bottom-up assembly, and top-down lithography [46,47,48]. These techniques enable precise control over the size, shape, and composition of nanomaterials, which can be tailored to specific applications such as electronic devices, drug delivery systems, solar cells, and batteries. Examples of organic-based nanomaterials include liposomes, micelles, dendrimers, and cyclodextrins. Dendrimers are a special type of organic-based nanomaterial with highly branched, tree-like molecules that have been developed specifically for biomedical applications, such as drug delivery and imaging [49,50,51]. Nanocellulose, which includes microfibrillated cellulose (MFC), nanocrystalline cellulose (NCC), and bacterial cellulose (BC), is an organic nanomaterial derived from natural polymers in wood/plants and bacterial strains, respectively. MFCs and NCCs are mostly used as reinforcement materials for enhancing the properties of polymer-based composites, whereas BC has applications mainly in biomedicine and water filtration treatment. Generally, nanocellulose can be obtained through various processes, such as mechanical, chemical, or enzymatic treatment [52,53].

#### 1.1.2. Inorganic Nanomaterials

Inorganic-based nanomaterials consist of inorganic elements or compounds synthesized at the nanoscale. These materials can be classified as metal or ceramic-based nanomaterials. Common metal-based nanomaterials include silver (Ag), copper (Cu), gold (Au), aluminum (Al), zinc (Zn), and lead (Pb) nanoparticles. Ceramic or metal oxide-based nanomaterials include silica (SiO_2_), copper oxide (CuO_2_), iron oxides (Fe_2_O_3_ and Fe_3_O_4_), titanium oxide (TiO_2_), and magnesium aluminum oxide (MgAl_2_O_4_) [40,54]. Inorganic-based nanomaterials have been successfully incorporated into polymer matrices to produce nanocomposites with enhanced properties for various applications, such as food packaging, coatings, and biomedicine, due to their unique optical, electrical, and other properties [55]. Nanoclay can also be classified as an inorganic-based nanomaterial derived from clay minerals, which are processed into nanometer-sized particles. Clays are naturally occurring minerals with high mechanical performance, good thermal stability, and environmental friendliness. Nanoclay particles such as montmorillonite, kaolinite, and halloysite, have been successfully used to enhance the performance of many polymer matrices to develop nanocomposite materials for various applications [56,57].

#### 1.1.3. Carbon-Based Nanomaterials

**Figure 1:**
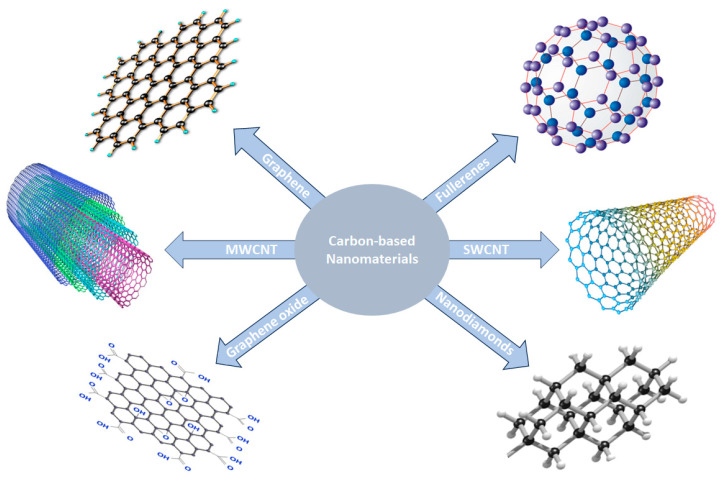
Structures of various carbon-based nanomaterials.

Carbon-based nanomaterials are a special type of nanomaterial that comes in different forms or allotropes and contains carbon atoms arranged in unique structures at the nanoscale level [58,59,60,61,62]. These materials have various shapes and sizes, depending on the type, and include carbon nanotubes, graphene, fullerenes, graphene oxides, and many others. Figure 1 shows the structural arrangement and shapes of different types of carbon-based nanomaterials. Due to the inherent attributes of these carbon-based nanomaterials, which include high electrical conductivity, high chemical stability, acceptable aspect ratio, biocompatibility, and exceptionally high mechanical properties, they have been recognized as promising materials for many critical applications such as biomedicine, biosensors/detectors, energy storage and conversion, functional composite development, surface coating, and environmental remediation [63].

#### 1.1.4. Graphene (G)

Graphene is a recently discovered carbon material with exceptional properties. It consists of a single layer of graphite with excellent mechanical, electrical, and thermal properties, surpassing those of traditional engineering materials such as metals and ceramics. Graphene consists of sp^2^ carbon atoms organized in a hexagonal lattice, forming a two-dimensional (2D) layer. It has an exceptional surface area (2630 m^2^/g), remarkable elastic modulus (1 TPa), high conductivity (intrinsic carrier mobility of 2,000,000 cm^2^/V), impressive optical transparency, and thermal conductivity (5000 W/mK) [64,65,66]. It appears to be a building block for other carbon-based nanomaterials such as carbon nanotubes and fullerenes. It has been described as one of the most promising materials for future applications because of its combined flexibility and excellent properties [67]. Graphene, often regarded as the thinnest material, also boasts remarkable strength. While steel typically exhibits a breaking strength ranging from 250 to 1200 MPa, equivalent to 0.25–1.2 × 10^9^ N/m^2^, if hypothetically drawn into sheets as thin as graphite (approximately 3.35 Å), its breaking strength would be reduced to a mere 0.084–0.40 N/m. In contrast, graphene exhibits an impressive breaking strength of 42 N/m, making it approximately 100 times stronger than steel [68]. It is highly impermeable to gases and liquids, making it a useful reinforcement material for nanocomposites by creating an interfacial barrier against gases and moisture absorption when added to a polymer matrix. The suitability or applicability of graphene as a potential reinforcement material in nanocomposite development has been studied by several researchers [69,70]. Consequently, graphene-based nanocomposites exhibit excellent mechanical and electrical properties and can be used in various applications, including electronics, energy storage devices, sensors, and biomedicine [67].

#### 1.1.5. Graphene Oxide (GO)

Graphene oxide (GO) is a derivative of graphene that consists of oxygen-containing functional groups, such as hydroxyl, epoxy, and carboxyl groups, attached to the graphene surface [71,72]. It is a two-dimensional material containing a single atomic layer arranged in a hexagonal lattice. The oxygen-based functional groups in graphene oxides make them highly hydrophilic and easily dissolvable in many polar solvents, such as water and organic solutions. In addition, it can be easily functionalized by attaching other molecules or nanoparticles to its surface. Graphene oxides are produced from a crystalline form of carbon (graphite) with strong oxidizing agents such as potassium permanganate or sodium nitrate [73,74]. It has a large surface-area-to-volume ratio and excellent thermal stability and chemical reactivity. GO has been widely used as a reinforcement material in composite production, catalysis, biomedical engineering, wastewater treatment, and environmental remediation [75,76,77,78]. Reports have shown that the presence of oxygen-containing functional groups in GO structures slightly affects its properties, such as electrical conductivity and mechanical strength, compared to those of pure graphene [79,80].

#### 1.1.6. Carbon Nanotubes (CNTs)

Carbon nanotubes are cylindrical structures consisting of rolled-up sheets of graphene in a two-dimensional form with a hexagonal lattice pattern. They were first discovered by Japanese scientist Sumio Iijima in 1991, who first observed them in the soot of an arc discharge between two graphite electrodes and clearly described their formation [81,82,83]. Following their discovery, CNTs have attracted much scientific interest due to their unique physical and chemical characteristics. They are strong and stiff yet flexible, with excellent thermal and electrical conductivity, making them ideal materials for sensitive applications [84]. CNT-based nanocomposites are widely used across various industries because of their exceptional specific strength, which exceeds that of many traditional engineering materials. This strength, combined with their lightweight nature, makes them ideal for applications where both durability and weight reduction are critical. In the automotive and aerospace industries, nanocomposites contribute to the development of lightweight yet strong components like body panels, chassis parts, and aircraft structures, enhancing fuel efficiency, safety, and overall performance. In the biomedical field, CNT-based nanocomposites are being explored for advanced medical implants and devices due to their biocompatibility, strength, and electrical conductivity. They are used in bone scaffolds, artificial joints, and targeted drug delivery systems, offering better mechanical support and improving treatment effectiveness. The sports industry also benefits from these materials, which are used to create high-performance equipment such as tennis rackets, golf clubs, and protective gear. Their superior strength-to-weight ratio allows for lighter, more responsive, and durable equipment, enhancing athletic performance and safety. Additionally, CNT-based nanocomposites are popular in the electronics industry for developing flexible electronics, sensors, and conductive films, as well as in energy storage devices like batteries and supercapacitors, where they improve energy density, efficiency, and charge/discharge rates. Depending on the arrangement of the graphene sheets within the material, CNTs can be classified as either single-walled (SWCNTs) or multi-walled (MWCNTs). SWCNTs consist of a single layer of graphene rolled into a tube to form a cylindrical structure. They have different diameters and chirality, which affect their physical and chemical properties. On the other hand, MWCNTs consist of multiple layers of graphene sheets rolled into nested tubes [22,85]. Reports have shown that the properties of MWCNTs largely depend on the number and arrangement of graphene layers in the structure. SWCNTs typically have diameters ranging from 0.7 to 2 nm and lengths that can vary from several microns to a few millimeters. The manner in which the graphene sheet is rolled determines the chirality of the nanotube, which is characterized by the chiral angle between the carbon hexagons and the tube’s axis. This chirality, along with the diameter, influences the nanotube’s electrical conductivity [86]. Generally, CNTs have been widely studied for their unique electrical, mechanical, and optical properties and have potential applications in electronics, energy, and materials science [84,87].

#### 1.1.7. Fullerene (F)

Fullerene is a class of carbon-based nanomaterials that was initially discovered in 1985 by a research team led by Harold Kroto, Robert Curl, and Richard Smalley. Their pioneering work on fullerenes earned them the 1996 Nobel Prize in Chemistry after 11 years of research. Fullerenes consist of carbon atoms organized into spherical or ellipsoidal molecules, resembling a soccer ball, as shown in Figure 1. Since their discovery, fullerenes have been the subject of continual and expanding research areas, which have sparked the creation of various novel and sophisticated materials for biomedical applications. In addition to their role in medication systems, fullerenes are also well known for their potential as superconductors and their capacity to form stable compounds with other molecules [88,89]. The versatile nature of fullerenes has led to their wide exploration in diverse fields, contributing significantly to advancements in science and nanotechnology. 

## 2. Recent Advances in Nano-Enhanced Composites

Nanomaterials have emerged as a promising class of materials due to their unique properties at the nanoscale, characterized by a high surface area-to-volume ratio and quantum effects [90]. These exceptional properties have significantly driven the use of nanomaterials to enhance the mechanical, electrical, thermal, and optical characteristics of nanocomposite materials, particularly polymer-based nanocomposites, including both biopolymers and petroleum-derived polymers. Nanocomposites with enhanced performance are highly desirable for diverse industrial applications, such as electronic devices, energy storage, biomedical devices, and environmental remediation. The growing demand for advanced composite materials has led to increased interest in developing nanocomposites with novel characteristics by incorporating nanomaterials like graphene, carbon nanotubes, and metal nanoparticles into composite structures [91]. These nanomaterials impart novel functionalities, significantly improving material properties such as mechanical strength, electrical conductivity, and thermal stability [92]. Nanomaterials such as graphene possess a high theoretical surface area, enabling better interfacial interactions with composite components, resulting in enhanced mechanical and electrical performance [93]. Similarly, fullerene-reinforced polymer matrix nanocomposites have been shown to improve electrical conductivity, mechanical strength, and thermal stability, making them ideal for energy storage applications such as supercapacitors [94]. Fullerenes offer an economical alternative to other carbon-based nanomaterials, such as carbon nanotubes and graphene, due to their abundant availability and lower cost [95].

Jani et al. [96] highlighted the current progress in the use of zero-dimensional (0D) carbon-based nanomaterials (such as fullerenes and carbon dots) in polymer-based nanocomposites for water purification/treatment. According to their findings, the incorporation of fullerenes and carbon dots into various polymer-based nanocomposites enhances interfacial membrane properties, leading to better water permeability, separation efficiency, and antifouling performance. Similarly, Ng et al. [97] reported the membrane performance of polymer-based nanocomposites for water purification using graphene oxide (GO) and its derivatives as nanofillers. This study showed that the introduction of GO nanofillers into a polymer matrix can significantly alter membrane morphology, surface wettability, and functional groups, resulting in enhanced wastewater treatment performance. Wang et al. [98] developed a carbon nanotube/polyurethane composite for efficient electromagnetic interference shielding. The results showed that 5.0 wt.% CNT loading in the X-band (8.2–12.4 GHz) resulted in a shielding effectiveness of 30.7 dB. In addition, at 2.0 wt.% CNT content, the developed composite displayed healable tensile properties with an elongation at break of 571% ± 31% and a self-healing efficiency of 89.2%.

In the field of biopolymers, research is gaining attention due to their environmental sustainability and potential to reduce reliance on fossil fuels [99]. Biopolymers, derived from natural resources such as corn starch, cellulose, and sugarcane, present an eco-friendly solution to the challenges of plastic waste and fossil fuel depletion [100,101]. However, biopolymers or bioplastics often exhibit limitations in mechanical strength, thermal stability, and barrier properties. Their inherent permeability to gases and vapor has driven increased research efforts aimed at enhancing their performance for sustainable applications, such as food packaging [34]. Incorporating nanomaterials into bioplastics has proven to be an effective strategy for addressing these limitations. Nanomaterials like nanocellulose, nanoclays, graphene, and carbon nanotubes enhance the mechanical strength, thermal stability, and overall performance of bioplastic-based nanocomposites, making them suitable for various applications, including packaging, medicine, and electronics [101]. The addition of nanoparticles can greatly enhance the mechanical and thermal properties of starch-based bioplastics [102]. Xie et al. [103] developed a simple method to produce high-performance starch-based bioplastics (SBP_SCZn−1.5_) using 2,2,6,6-tetramethylpiperidine 1-oxy-oxidized cellulose nanofibers (TEMPO-CNF) and zinc oxide nanoparticles (ZnO), resulting in a robust chemical and physical double crosslinked network. These bioplastics demonstrated excellent mechanical strength (24.54 MPa), along with water and heat resistance, as well as biodegradability. Their outstanding properties make them promising alternatives to petroleum-based plastics for applications in packaging and daily necessities.

Martínez-Rubio et al. [104] developed a new nanocomposite based on the biopolymer PHBV, incorporating 3% calcined hydrotalcite (CHT) and cloisite 20A (C20A). The addition of CHT improved surface smoothness, crystallinity, and lowered the degradation temperature. X-ray diffraction and rheological analysis confirmed effective polymer-additive interactions and enhanced viscoelastic behavior. Meanwhile, structures containing C20A showed greater thermal stability but weaker mechanical properties compared to those with CHT. Ancy et al. [105] developed cassava starch-based bioplastics with different plasticizer ratios, with the optimal 1:0.05 ratio demonstrating the highest tensile strength. Zinc oxide and silver nanoparticles synthesized using Hibiscus and Tulsi leaves improved the bioplastics’ durability, thermal stability, and biodegradability across various environments. These nanoparticle-infused bioplastics hold promise for eco-friendly commercial applications like disposable bags, combining strength, stability, and faster degradation. Srisuwan et al. [106] developed high-molecular-weight poly(L-lactide)-*b*-poly (ethylene glycol)-*b*-poly(L-lactide) (PLLA-PEG-PLLA), a flexible, biodegradable bioplastic with potential for food packaging but lacking antibacterial properties. The addition of zinc oxide nanoparticles (nano-ZnOs) improved its crystallization, tensile strength, UV-barrier, and antibacterial properties. However, adding excessive nano-ZnO (>2 wt.%) led to reduced performance. The nanocomposite films also demonstrated effective antibacterial activity against *Escherichia coli* and *Staphylococcus aureus*, establishing nano-ZnOs as multifunctional fillers for enhanced food packaging. Two series of poly (propylene 2,5-furandicarboxylate)-block-poly (tetramethylene oxide) (PPF-b-F-PTMO) nanocomposites were synthesized via in situ polymerization. The addition of 1D-type nanoparticles, such as carbon nanofibers (CNFs) and halloysite nanotubes (HNTs), and 2D-type nanoparticles, including graphene nanoplatelets (GNPs) and organoclay (C20A), enhanced crystallinity and tensile modulus, though a decrease in limited viscosity was observed [107].

Nanomaterials, such as carbon nanotubes, graphene, metal nanoparticles, and nanoclays, have demonstrated the ability to significantly improve the strength, durability, and functionality of polymer-based nanocomposites [108]. These enhancements are achieved through mechanisms such as increased interfacial interactions and better load transfer. Recent studies have shown that incorporating nanomaterials can lead to remarkable improvements in material properties, such as higher tensile strength, better thermal stability, improved electrical conductivity, and enhanced barrier properties, making them suitable for a range of advanced applications in industries like electronics, aerospace, biomedical devices, and packaging [109,110]. The use of nanomaterials to enhance the performance of polymer-based nanocomposites, particularly the mechanical, thermal, electrical, and optical properties, is explored in the following sections, showcasing key recent advancements from the literature.

### 2.1. Mechanical Properties

Nanocomposites exhibit superior mechanical properties that surpass those of conventional composite materials. They offer a combination of attractive properties, such as high specific strength and stiffness, toughness, and wear resistance, because of the synergistic effects of the nanofillers and the matrix components [111]. Understanding the strengthening mechanisms of nanomaterials during nanocomposite development is crucial for optimizing their performance and unlocking their full potential in diverse applications, including the transportation, biomedical, and construction sectors. Several reports have demonstrated the remarkable ability of nanomaterials to significantly enhance the mechanical performance of composite materials. The use of CNTs and other carbon-based nanomaterials has been widely studied due to their remarkable strength and stiffness [112,113]. It has been reported that the use of both ex-situ CNTs and in-situ γ-alumina nanoparticles can lead to the development of high-performance metal matrix composites, especially aluminum matrix nanocomposites (Al-MMCs) [114]. Ma et al. [115] developed nanocomposites consisting of carbon nanotubes (CNTs) and a graphene (G) hybrid aerogel as a conductive network in a polydimethylsiloxane matrix to enhance the piezoresistive performance of the multiscale hybrid nanocomposites. CNTs were grown on graphene sheets through chemical vapor deposition using an in-situ processing approach. The hybrid aerogel improved the interfacial interaction between the conductive network and polymer matrix, exhibiting high sensitivity and an outstanding linear dependence on the stress applied to the material. 

**Figure 2:**
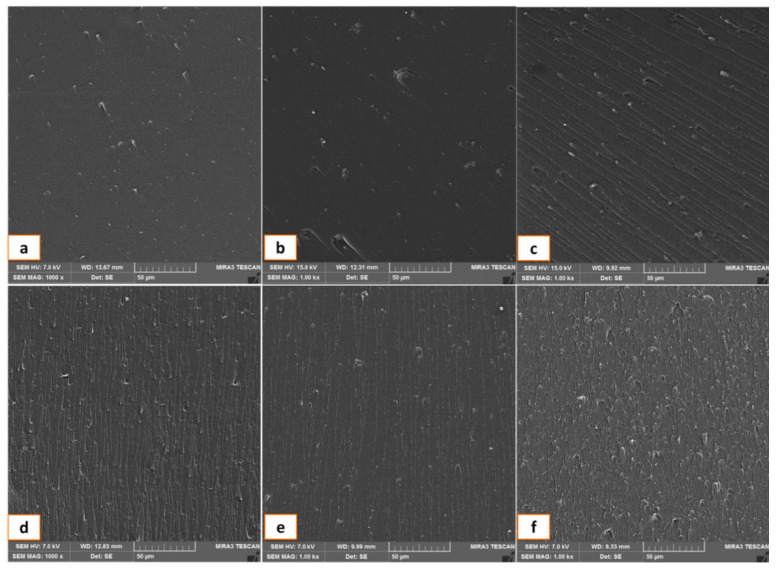
FE-SEM micrographs of (**a**) pure UPE, (**b**) UPE/GO, (**c**) UPE/GN, (**d**) UPE/25GO:75NC, (**e**) UPE/50GO:50NC, and (**f**) UPE/75GO:25NC [116].

**Figure 3:**
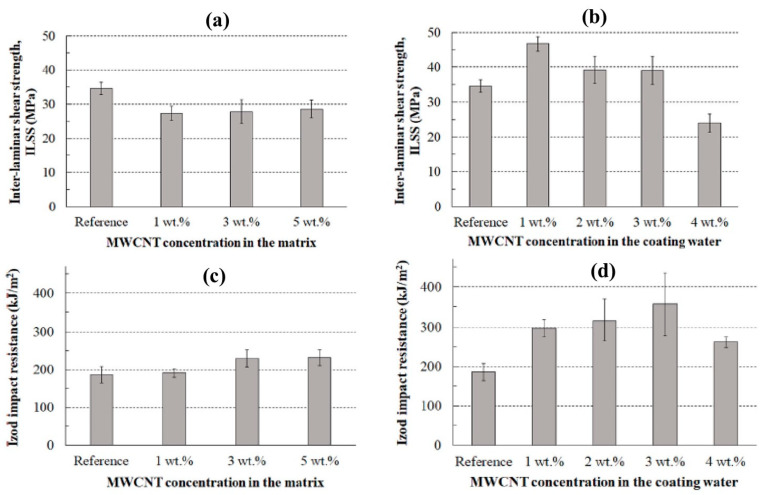
(**a**) ILSS for the direct mixing of MWCNTs in resin, (**b**) ILSS for MWCNTs anchored on CF, (**c**) impact resistance for the direct mixing of MWCNTs in resin, and (**d**) impact resistance for MWCNTs anchored on CF [109].

Farshidfar et al. [116] used hybrid nanofillers (graphene oxide/nanoclay) in an unsaturated polyester resin (UPE) to improve the mechanical and thermal properties of the hybrid nanocomposites and reported significant improvement in properties due to the homogeneous dispersion of the nanoparticles in the polyester resin. The results of the study also indicated the positive synergistic effects of graphene oxide (GO) and nanoclay (NC) at unequal ratios of (25GO:75NC and 75GO:25NC) with well-dispersed fillers uniformly distributed within the polymer substrate and a minimum degree of aggregation, as observed in the FE-SEM micrograph in Figure 2d,f. The effect of graphene nanoplatelets (GNPs) on the mechanical and thermal properties of recycled polycarbonate nanocomposites was also studied under various filler contents and other processing parameters. The results showed that Young’s modulus and yield strength of the composites were enhanced with increasing GNP loading, as well as the thermal properties of the nanocomposites [117]. Cheon and Kim [109] investigated the influence of MWCNTs on the impact resistance and interlaminar shear strength (ILSS) enhancement of carbon fiber-reinforced polyamide 6 composites. This study compared the effectiveness of MWCNTs anchored on carbon fibers (CF) with direct mixing of MWCNTs in thermoplastic resin. The results, shown in Figure 3, demonstrate that MWCNTs anchored on the carbon fibers improved the interfacial interaction between the fiber and the matrix without increasing the viscosity of the resin. This improvement significantly enhanced both the impact resistance and ILSS of the composites compared with the direct mixing approach.

**Figure 4:**
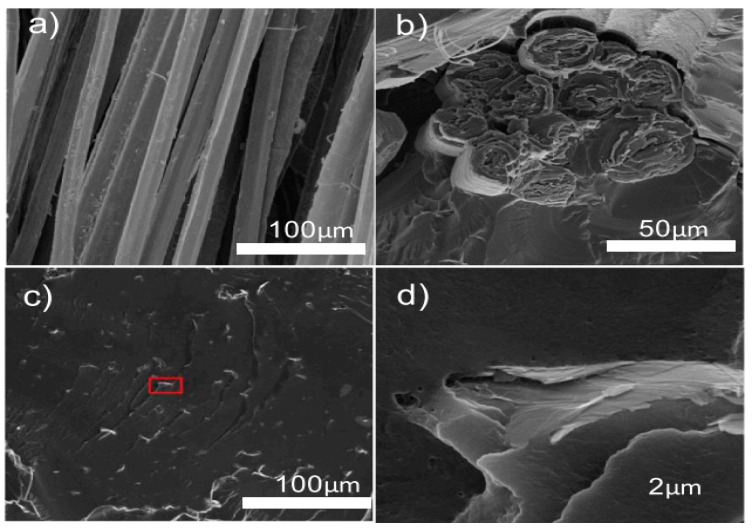
SEM micrographs: (**a**) surface of pristine hemp yarns, (**b**) cross-section of hemp-WPU/H25GNP/0.5%, (**c**) H25GNP distribution in WPU/H25GNPs/0.5%, and (**d**) H25GNP-to-WPU interface in WPU/H25GNP/0.5% [118].

Abbass et al. [118] investigated the impact of nanocoating on the mechanical performance and environmental resistance of flax and hemp fibers in masonry retrofitting applications. A nanocomposite coating comprising graphene nanoplatelets (GNPs) and water-borne polyurethane (WPU) was applied to the fibers. The coated fibers exhibited significant enhancements in their tensile properties with low water absorption. The tensile strength and elastic modulus of the coated hemp yarns were improved by 120% and 163%, respectively, at an optimal weight percentage of 0.5 wt.% GNPs compared with the uncoated yarns. This improvement is attributed to the uniform distribution of nanoplatelets within the polymer matrix, resulting in effective wetting and enhanced interfacial interactions with the polymer matrix, as shown by the SEM image in Figure 4d. 

**Figure 5:**
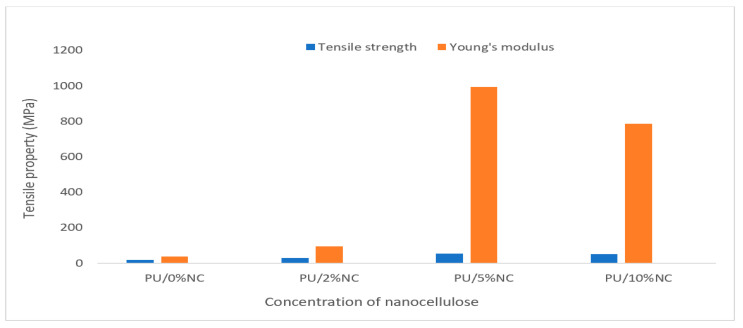
Mechanical properties of pure polyurethane and nanocellulose-embedded composites [120].

Katagiri et al. [119] studied the effects of cellulose nanofiber (CNF) content on the impact properties of carbon fiber-reinforced (CFRP) epoxy composites. This study compared CNFs derived via 2,2,6,6-tetramethylpiperidine-1-oxylradical (TEMPO)-mediated oxidation, followed by mechanical fibrillation, and CNFs obtained directly by mechanical fibrillation from hardwood pulp without TEMPO-mediated oxidation. The results showed that the impact energy absorbed by the CFRP specimen increased with increasing CNF content up to a maximum of 21% by the CNF dispersion layers. Cherian et al. [120] developed polyurethane-based (PU) nanocomposites using nanofibers isolated from pineapple leaves as reinforcement. The study showed that the addition of 5 wt.% cellulose nanofibrils to PU yielded nearly 300% and 2600% increases in the tensile strength and stiffness, respectively (Figure 5). The authors concluded that the developed composites can potentially be used to fabricate various medical implants for biomedical applications. Ansari et al. [121] developed nanostructured biocomposites by incorporating a nanocellulose network in an unsaturated polyester (UP) resin and reported a substantial increment in the glass transition temperature (Tg), which increased with 45 vol% NC content. The modulus and strength of the composite were three times higher, while both the ductility and apparent fracture toughness were doubled at 45 vol% NC compared with UP.

Lee et al. [122] synthesized a novel and robust nonwoven sisal fiber preform using bacterial cellulose (BC) as a binder in acrylate-epoxidized soybean oil (AESO) to enhance performance. This approach significantly improved the storage modulus of the composite and enhanced the fiber–matrix stress transfer.

**Figure 6:**
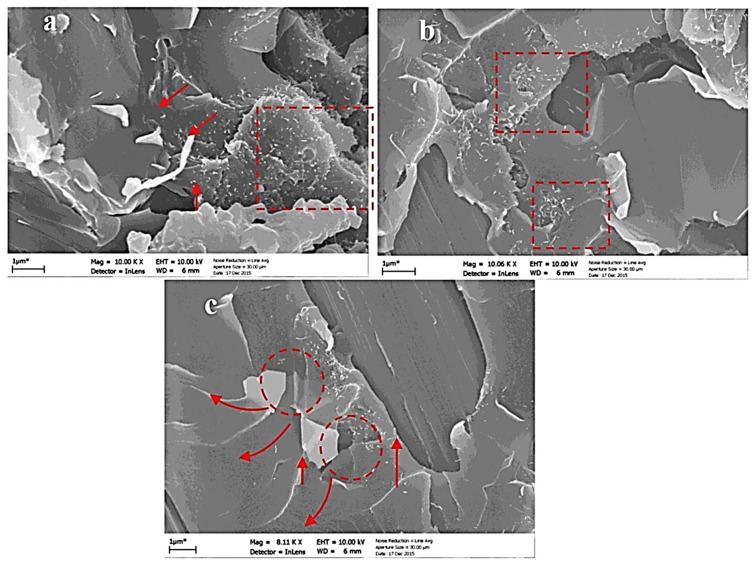
SEM images showing the failure mode and strengthening mechanisms of hybrid nanofillers in composites: (**a**) shows the presence of both fillers within the matrix material, highlighted by the square box, (**b**) displays MWCNTs attached to GNPs, as indicated by the red-square boxes, and (**c**) illustrates the pull-out behavior of MWCNTs/GNPs, with the bifurcation caused by GNPs circled in the images [123].

Kostagiannakopoulou et al. [123] investigated the synergistic effect of two carbon-based nanofillers, few-layered graphene nanoplatelets (GNPs) and multi-walled carbon nanotubes (MWCNTs), on the interlaminar fracture toughness of carbon fiber-reinforced polymer composites (CFRP). Two experimental tests were conducted. In the first experiment, 0.5 wt.% GNPs and 0.5 wt.% MWCNTs were used to modify the polymer matrix. In the second experiment, the concentration of GNPs was held constant, whereas the concentration of MWCNTs was increased to 1 wt.%. The addition of 0.5 wt.% GNPs and 1 wt.% MWCNTs improved the mode I fracture toughness (G_IC_) by 45% and mode II (G_IIC_) by 25%. Figure 6 shows the SEM micrographs of the fracture surfaces illustrating the synergistic effects and strengthening mechanisms of the hybrid nanofillers in the composite.

**Figure 7:**
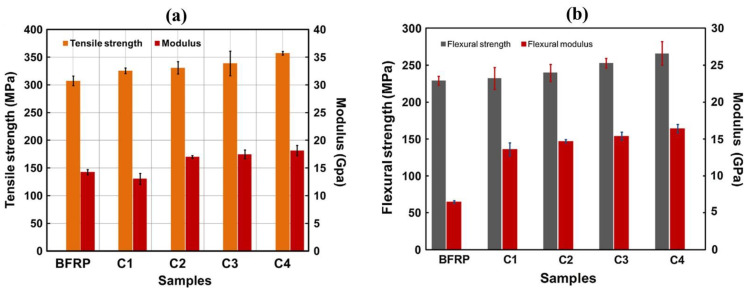
Mechanical performance of TM-modified BFRP composite laminates: (**a**) average tensile strength and modulus and (**b**) average flexural strength and modulus [124].

**Figure 8:**
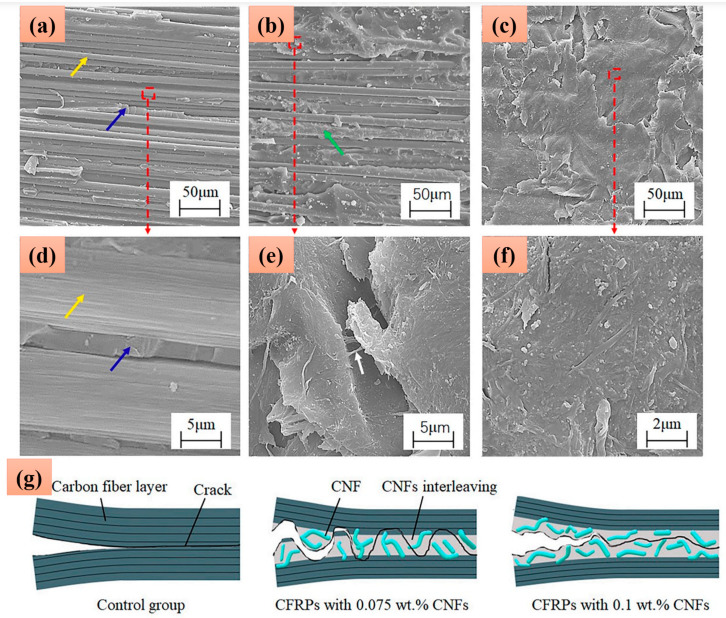
SEM images of CFRPs fractured by DCB tests: (**a**,**b**) control group, showing clean fiber pull-out (indicated by yellow arrows) and brittle epoxy failure (indicated by blue arrows), (**c**,**d**) 0.075 wt.% CNFs, showing CNF bridging (indicated by green arrows) and fiber pull-out (indicated by white arrows), (**e**,**f**) 0.1 wt.% CNFs show CNF coverage with delamination, and (**g**) schematic of crack propagation [125].

Subagia et al. [124] investigated the influence of tourmaline (TM) micro/nanoparticles at varying concentrations (Table 1) on the tensile and flexural properties of basalt fiber-reinforced polymer (BFRP) composites. Their findings indicated that the addition of 1 wt.% TM, along with a surfactant, resulted in optimal tensile and flexural strengths, showing an improvement of approximately 16% compared with the neat basalt/epoxy composite. Moreover, the study demonstrated significant increases of 27.4% and 153.3% in the tensile and flexural moduli, respectively, as shown in Figure 7a,b. This enhanced performance was attributed to the effective dispersion of TM particles in the epoxy matrix at a 1 wt.% concentration with the surfactant, which promoted strong interfacial bonding between the basalt fibers (BFs) and the epoxy resin, thereby improving load transfer and overall composite performance. Zhu et al. [125] investigated the impact of cellulose nanofiber (CNF) content on the interlaminar fracture toughness and damping properties of carbon fiber-reinforced polymer composites (CFRPs). CNF films were developed and infused between the carbon fibers in various stacking sequences to assess the loss factors of the resulting composites. The results revealed that the incorporation of 0.075 and 0.05 wt.% CNFs led to 22% and 25% improvement in the mode I and II interlaminar fracture toughness of CFRPs, respectively. Conversely, the addition of 0.25 wt.% CNFs increased the loss factor by 31%. However, this enhancement in damping properties was accompanied by a slight reduction in the tensile and flexural properties of the CNF-interleaved CFRP. The SEM image presented in Figure 8 elucidates the strengthening mechanism of the CNF-interleaved CFRP and the fracture surface morphology of the composites.

### 2.2. Electrical Properties

Nanomaterials are known for their attractive electrical characteristics, which are attributed to their unique features at the nanoscale level. The incorporation of nanoparticles, nanowires, or nanotubes into the composite matrix enables precise control over electrical conductivity, resistivity, and dielectric properties [126]. Nanomaterials possess excellent electrical properties mainly due to the quantum confinement effect at the nanoscale level [127]. The energy required to charge electrons is confined within a limited space, leading to an increased number of charged electrons with effective mobility of the charged carriers, thereby significantly improving the electrical performance. Composites reinforced with nanomaterials, particularly carbon-based nanomaterials such as graphene, CNTs, and fullerenes, offer lightweight advantages, efficient electrical characteristics, and enhanced functionality [128]. These properties make them suitable for various applications, including electronics, sensors, and energy storage devices. These nanomaterials are recognized for their ability to enhance the dielectric properties of nanocomposites by mitigating partial discharge (PD) and reducing the accumulation of space charge within the composite structures [129]. The unique characteristics and interactions of nanomaterials at the interface with the polymer matrix lead to improved insulation performance and stability in electrical applications.

**Figure 9:**
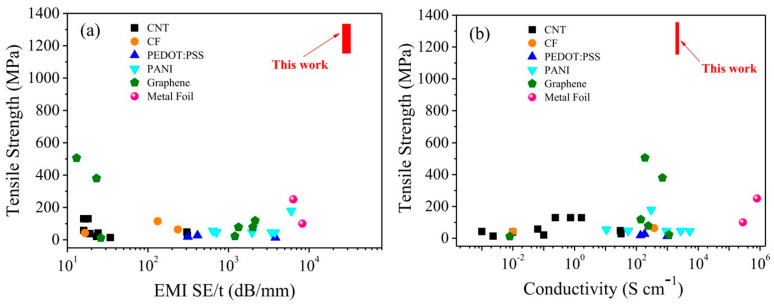
Property comparisons between the present shielding material and previous ones involving the use of carbon nanotubes (CNT), carbon fibers (CF), poly (3,4-ethylenedioxythiophene): poly (styrenesulfonate) (PEDOT: PSS), polyaniline (PANI), graphene, and metal foils: (**a**) tensile strength versus EMI SE per millimeter thickness (t) and (**b**) tensile strength versus electrical conductivity [130].

Conductive polymer-based nanocomposites, formed by integrating carbon nanomaterials such as graphene or CNTs, have garnered attention as effective materials for electromagnetic radiation protection due to their exceptional electrical conductivity, flexibility, and lightweight characteristics. These nanocomposites offer superior electrical properties, mechanical strength, and electromagnetic interference (EMI) shielding abilities. Wu et al. [130] developed a 1-mm thick CNT/polymer composite film with high tensile strength (1250 MPa) and excellent EMI shielding effectiveness (30 dB from 1 GHz to 18 GHz), outperforming previous findings, as shown in Figure 9, which compares tensile strength, EMI shielding effectiveness, and electrical conductivity with related studies. Dubey et al. [131] conducted a numerical analysis using Ansys HFSS to evaluate the EMI shielding effectiveness (SE) of conductive polymer nanocomposites (CPNC) with carbon nanotubes (CNTs) in the X-band. The study found that increasing CNT weight percentage and material thickness significantly enhances EMI SE, with a maximum SE of 180 dB observed in a hollow cylindrical CPNC. Additionally, a 107% improvement in absorption was achieved in a 4 mm pallet-shaped CPNC compared with a 1 mm thickness.

**Figure 10:**
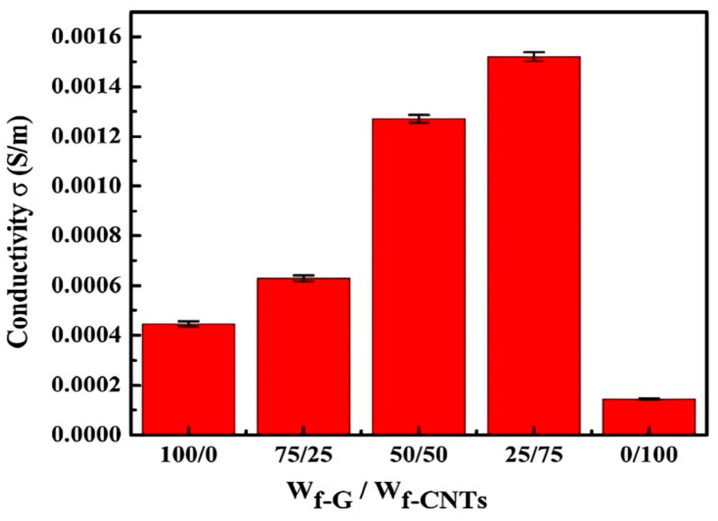
Electrical conductivity of PES-filled hybrid fillers at different weight ratios of graphene/CNTs for a filler content of 5 wt.% [132].

**Figure 11:**
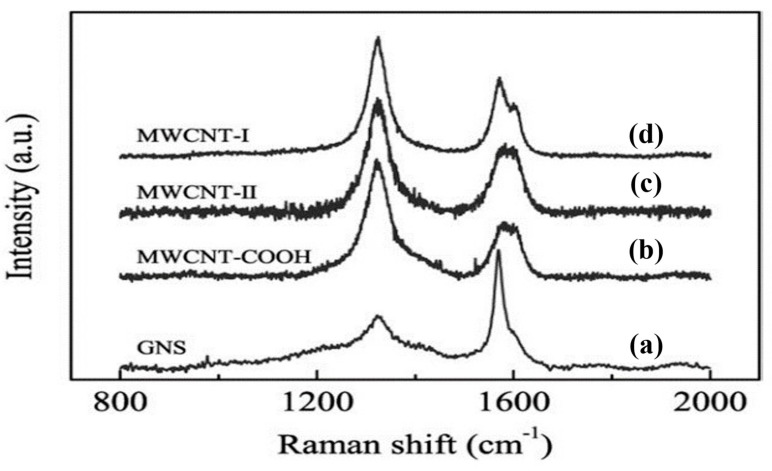
Raman spectra: (**a**) GNS, (**b**) functionalized MWCNT–COOH, (**c**) MWCNT-II, and (**d**) MWCNT-I [133].

Zhang et al. [132] explored the individual and synergistic effects of graphene and CNT-mixed fillers on the electrical conductivity of polyether sulfone (PES) polymer composites. They reported that the electrical conductivity of 5 wt.% graphene/PES composites was 5.82 × 10^−4^ S/m, surpassing that of CNTs/PES composites (1.43 × 10^−4^ S/m). Moreover, the graphene–CNT (graphene/CNTs = 1:1)/PES composite exhibited a lower percolation threshold (0.22 vol%) than the graphene/PES composites, with electrical conductivities 2.2 and 8.9 times higher than those of the 5 wt.% graphene/PES composite and CNTs/PES composite, respectively. This enhancement was attributed to the high aspect ratio and synergistic effects of the mixed fillers, which facilitate the co-dispersion of graphene and CNTs and establish a more efficient 3D percolation network. In Figure 10, it is evident that the electrical conductivity of the composite steadily increases as graphene is substituted by CNTs, exhibiting notable optimal performance at a graphene/CNT ratio of 1:3. Moreover, nanocomposites containing only one of the two fillers demonstrated diminished electrical conductivity. He et al. [133] investigated the influence of CNTs and Graphene Nanosheets (GNSs) functionalization on the electrical properties of epoxy-based nanocomposites. Three different types of Multiwalled Carbon Nanotubes (MWCNTs) were used, namely, MWCNT-I (pristine, purity > 95%, diameter = 20–30 nm, length ≈ 20 μm), MWCNT-II (pristine, purity > 95%, diameter = 30–50 nm, length ≈ 20 μm), and MWCNT–COOH (functionalized, diameter = 30–50 nm, length ≈ 20 μm). Their findings showed that the pristine MWCNT)/epoxy composite exhibited higher conductivity (0.176 S/m), approximately 11 times that of the pure epoxy (1.2 × 10^−12^ S/m) and 7 times that of the functionalized MWCNT/epoxy (1.9 × 10^−8^ S/m). The Raman spectrum (Figure 11) indicated that the measured intensity of the highly reduced graphene nanosheets (GNSs) was much smaller than that of the pristine (MWCNT/epoxy) and functionalized (MWCNT–COOH/epoxy) composites. This discrepancy was attributed to the interaction between the graphene sheets and epoxy resin, which prevented electrons from hopping between them, thereby reducing the overall electrical conductivity of the GNS-based composite. From the Raman spectrum, the ratio of D to G peak intensities (I_D_:I_G_) was calculated to assess the structural imperfections in the composites. MWCNT-I showed the most intact cylindrical structure with an I_D_:I_G_ ratio of 1.54, whereas MWCNT-II and MWCNT–COOH exhibited higher ratios of 1.72 and 2.22, respectively, indicating more defects. This analysis facilitates the comparison of the structural characteristics of different CNTs and the impact of combining them with GNSs as fillers. In addition, the percolation threshold was noted at 0.2 wt.% for pristine MWCNTs and 0.3 wt.% for graphene in the epoxy composites.

**Figure 12:**
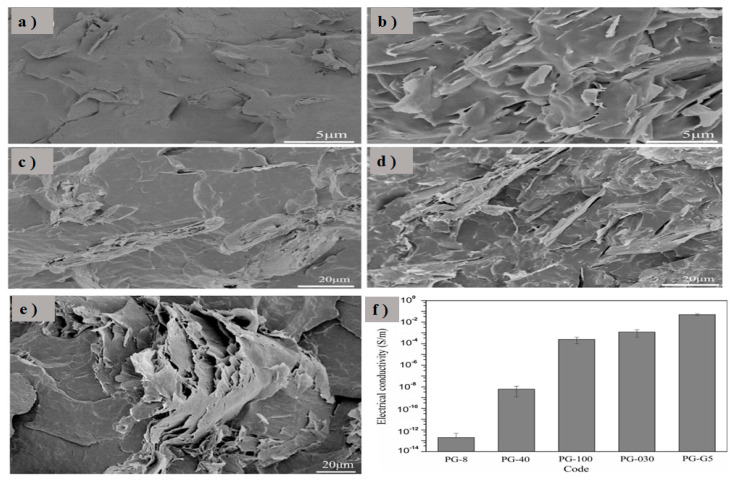
FESEM micrographs and electrical conductivity of PP/GNP nanocomposites with different sizes of GNPs at 12 wt.% content: (**a**) PG-8, (**b**) PG-G5, (**c**) PG-40, (**d**) PG-030, (**e**) PG-100, (**f**) electrical conductivity [134].

In a separate study conducted by He et al. [134], the impact of graphene nanoplatelets’ (GNPs) aspect ratio on the electrical conductivity of polypropylene (PP) filled with GNPs and their hybrid systems was investigated. The study examined five different aspect ratios of GNPs: 8 (PG-8), 400 (PG-40), 1000 (PG-100), 1300 (PG-030), and 1500 (PG-G5). PG-8, PG-40, and PG-100 had the same thickness of 100 nm but differed in diameter. The concentration of graphene was fixed at 12 wt.%. This study revealed that GNPs with a larger diameter and smaller thickness are more effective in forming a conductive network. In Figure 12b, better dispersion was observed for PG-G5 because of its lower thickness. Thicker layers typically result in a lower degree of exfoliation and dispersion in the polymer matrix. This SEM image further elucidated the enhanced conductive network formation shown in Figure 12f.

**Figure 13:**
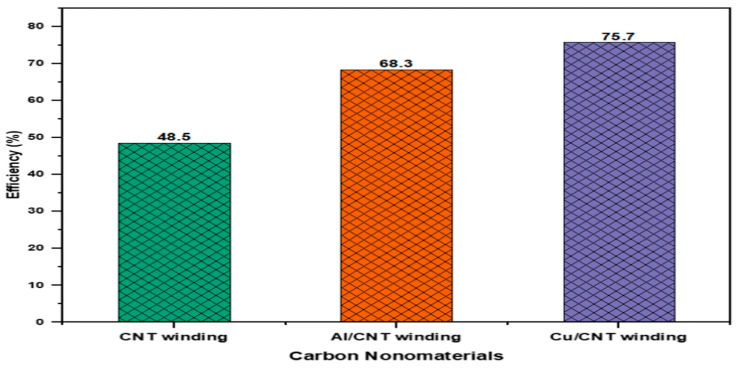
Average efficiencies of permanent magnet generator with various materials winding [135].

Hassan et al. [135] compared three CNT-based composites as potential winding materials for permanent magnet generators: CNT yarn, Al/CNT, and Cu/CNT. The authors analyzed the performance of these materials in terms of generated voltages, currents, and efficiencies using a finite element model simulated in the COMSOL Multiphysics software. Results indicated that the Cu/CNT composite wire generated the highest voltage (70 V) at 100 rpm, followed by Al/CNT (50.1 V) and CNT yarn (15.2 V). Figure 13 shows the average efficiencies of the permanent magnet generator with various materials winding. This study indicates that carbon nanomaterials have the potential to replace conventional metal-based windings in electrical machines, with Cu/CNT composite wire being particularly favorable compared with CNT yarn for winding applications.

### 2.3. Thermal Properties

Nanomaterials such as nanoparticles, nanofibers, nanotubes, and graphene offer greater opportunities to improve thermal conductivity, heat dissipation, and thermal stability in composite materials owing to their inherent and unique nanoscale properties. Nanomaterials such as carbon nanotubes (CNTs), graphene, and some metallic nanoparticles possess exceptionally high thermal conductivities. When these nanomaterials are integrated into a composite structure, they facilitate the easy generation of conductive pathways within the composite structure, leading to efficient transfer of heat throughout the composite [126,136]. This significantly enhances the thermal conductivity of the nano-based composites. The unique characteristics of nanomaterials offer versatility in composite design, allowing for the creation of tailored composite structures with the desired thermal properties. By controlling factors such as filler type, filler concentration, dispersion, and orientation, researchers can optimize thermal conductivity and thermal stability to meet specific application requirements [137]. In addition, managing heat dissipation in electronic devices often involves using thermal pastes, thermal pads, and thermal compensators. Thermal pastes, or greases, fill microscopic gaps between a heat source and a heat sink to improve thermal conductivity. Thermal pads are solid, flexible materials that ensure consistent heat transfer where pastes may not be practical. Thermal compensators, designed for devices with variable thermal loads, help maintain stability. Nanocomposite materials are commonly used in these applications to enhance thermal conductivity and mechanical properties, providing more efficient and reliable thermal management.

Ujah et al. [138] demonstrated that reinforcing aluminum (Al) with 4 wt.% CNTs via spark plasma sintering improved the thermal conductivity of the composite by 35%. This enhancement is attributed to the increased vibrational motion of electrons and phonons due to the introduction of CNTs, which elevates their scattering and resistivity. The study also reported significant improvements in tribological properties, suggesting that Al-CNT composites are promising for industrial applications requiring efficient heat dissipation and electrical conductivity. Ali et al. [139] reported a significant enhancement in the heat transfer ability of paraffin wax by incorporating nanographene (NG) for safety helmet design. The study showed that the addition of 3 wt.% NG significantly improved the thermal conductivity of the NG/paraffin composite by up to 146%, suggesting that the composite can dissipate heat more effectively, thereby improving helmet cooling and performance. Additionally, a 3% reduction in latent heat was observed in the NG-modified composite compared with pure paraffin wax. This slight decrease in latent heat suggests that while the NG/paraffin composite exhibits improved thermal conductivity, it may have a marginally reduced capacity for thermal energy storage. However, this trade-off is likely outweighed by the enhanced heat dissipation, making the material more effective for applications like safety helmets, where rapid heat transfer is critical for comfort and safety. Zhang et al. [140] used carboxymethylcellulose-boron nitride nanosheets (CMC-BNNS) to enhance the heat resistance of biodegradable poly (propylene carbonate) (PPC). By exfoliating and functionalizing hexagonal boron nitride (h-BN) with sodium carboxymethylcellulose (CMC-Na), improved thermal properties were achieved. The resulting composites exhibit superior heat resistance, thermal conductivity, and dynamic mechanical properties compared with PPC/BN composites, offering a promising strategy to address the limitations of biodegradable polymers in practical applications. Similarly, Bertolino et al. [141] presented a novel method for fabricating multilayer composite biofilms using halloysite nanotubes (HNTs) and sustainable polymers like hydroxypropyl cellulose and chitosan to improve thermal performance. By varying the hydroxypropyl cellulose/HNTs ratio, the thermal properties were analyzed through differential scanning calorimetry (DSC) and thermogravimetry (TG). The results demonstrate enhanced thermal stability and flame-retardant potential.

**Figure 14:**
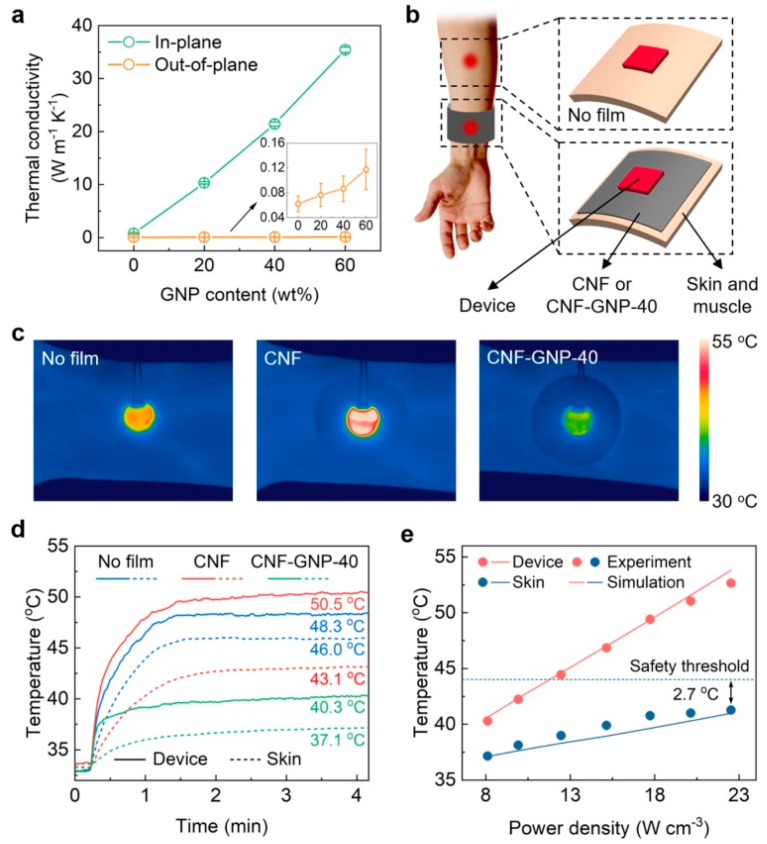
Thermal properties of CNF–GNP films: (**a**) in-plane and out-of-plane thermal conductivities of CNF–GNP composite films with different GNP contents, (**b**) schematic illustration of a wearable device with and without a film to dissipate heat, (**c**) infrared images of the three scenarios, (**d**) real-time temperature curves of the device and skin at the same power density. The solid and dashed lines represent the temperature of the device and skin, respectively, and (**e**) experimental and simulation results of the device and skin temperatures at different power densities [142].

Zhang et al. [142] developed high-performance, flexible, and lightweight nanocomposites using natural cellulose nanofiber (CNF) and graphite nanoplatelets (GNPs) derived from wood pulp and graphite powder, respectively. These films were fabricated using a simple bottom-up approach, involving solution mixing, vacuum filtration, and hot-pressing techniques. The composite films exhibited excellent flexibility and lightweight properties; alongside remarkable thermal conductivity of 21.42 W m^−1^ K^−1^ measured in CNF–GNP-40 films. The study also considered a wearable device and conducted a comparative experiment to assess the actual thermal management performance of the CNF–GNP-40 film. The experiment involved three scenarios: in the first, the wearable device was directly attached to the skin, while in the others, a film (CNF film and CNF–GNP-40 film separately) was interposed between the device and the skin (refer to Figure 14). The CNF–GNP-40 film demonstrated the capability to lower the temperature of both the device and the skin to only 40.3 °C and 37.1 °C, respectively (see Figure 14d), which are significantly lower and safer than in the former two scenarios. This study indicates that these composites could serve as valuable materials for various structural integrity.

Haghighi et al. [143] investigated the effect of various nanomaterials, including CuO, TiO_2_, Al_2_O_3,_ and graphene, on the thermal performance of a phase change material (PCM) using paraffin as a standard PCM. The results indicate that the nano-enhanced PCM exhibits remarkable thermal performance. A comparison among the investigated PCM-enhanced nanocomposites showed that the nanocomposites containing 2 wt.% TiO_2_, with an enthalpy of 179.88 J/g, and 1 wt.% graphene, with an enthalpy of 120.38 J/g, had the highest and lowest energy storage capacities, respectively, compared with unmodified paraffin. A similar study was conducted by AlOtaibi et al. [144], who reported the successful modification of paraffin wax, which was used as the standard PCM in this study, with multi-walled carbon nanotubes (MWCNTs) and various concentrations of TiO_2_ ranging from 3 wt.% to 7 wt.% at 2 wt.% intervals. The composites exhibited a significant increase in thermal conductivity by 100% at 5 wt.% of TiO_2_, along with the highest latent heat of enthalpy of 176 J/g recorded at 3 wt.% of TiO_2_. Furthermore, the developed nanocomposites demonstrated improved thermal stability after 15 thermal cycles compared with pure paraffin. Huang et al. [145] investigated the flammability and thermal stability of acrylonitrile–butadiene–styrene copolymers (ABS) to broaden their practical applications. In this study, they employed a graphene-derived flame retardant (Mo5/PN-rGO), which was designed by incorporating functional elements (phosphorus, nitrogen, and molybdate) onto graphene oxide nanosheets. The findings of the study revealed that the ABS nanocomposite containing 1.0 wt.% of Mo5/PN-rGO exhibited an increase in the glass transition temperature (Tg) by approximately 12 °C, whereas the onset thermal decomposition temperature was significantly delayed by approximately 21 °C. In addition, the developed ABS-based nanocomposites displayed a 20% reduction in total heat release and a 45% decrease in total smoke production compared with the pure ABS bulk material. Allahbakhsh et al. [146] explored the interfacial interactions and thermal performance of poly (ethylene trisulfide) (PETRS)/graphene oxide (GO) nanocomposites, modified with sodium dodecylbenzene sulfonate (SDBS) as a surfactant. Their findings indicate that the interactions between SDBS-modified GO and PETRS extend the melting process and degradation range of the resulting nanocomposites. Furthermore, the presence of SDBS-modified GO nanosheets noticeably increases the melting enthalpy of PETRS macromolecules.

### 2.4. Optical Properties

Nanomaterials play a crucial role in enhancing the optical performance of nanocomposites due to their unique tunable optical characteristics, quantum confinement effects, and high surface area-to-volume ratio [147]. These nanoscale materials can influence various optical properties of nanocomposites, such as absorption, emission, and scattering characteristics. Moreover, by controlling the size, shape, and surface chemistry of nanoparticles, it is possible to tailor these properties to meet specific requirements for optical applications. For instance, incorporating quantum dots or plasmonic nanoparticles can result in enhanced light absorption or emission, enabling advancements in areas such as solar cells, light-emitting diodes (LEDs), optical sensing, and optoelectronic devices like transparent electrodes, touchscreens, and smart windows [148]. Nanomaterials such as graphene oxide (GO), reduced graphene oxide (RGO), zinc oxide (ZnO), and their hybrids can significantly influence the optical behavior of nanocomposites. Ebrahimi Naghani et al. [149] investigated the linear and nonlinear optical performance of graphene oxide (GO) and reduced graphene oxide (RGO)-based zinc oxide (ZnO) nanocomposites and compared these with pure GO and RGO. Various characterization techniques, including Fourier transform infrared (FT-IR), ultraviolet-visible (UV-Vis) absorption, X-ray diffraction (XRD), and energy-dispersive X-ray spectroscopy (EDX), were used to characterize the synthesized nanocomposites. The results indicated that the nonlinear absorption coefficient value (β) increased from 5.3 × 10^−4^ (GO) to 8.4 × 10^−3^ cm/W (RGO-ZnO). In addition, the nonlinear refractive indices (n_2_) of GO, RGO, GO-ZnO, and RGO-ZnO were obtained as 10.9 × 10^−10^, 14.3 × 10^−10^, 22.9 × 10^−10^, and 31.9 × 10^−10^ cm^2^/W, respectively. Alshammari et al. [150] endeavored to develop nanocomposite materials for optoelectronic sensor applications by incorporating various ratios of graphitic carbon nitride (g-C_3_N_4_) nanosheets (0.0, 0.3, 0.6, and 1.0 wt.%) into PVC/PVP polymer nanocomposites. The results indicated that the optical absorbance and energy gaps of the PVC/PVP films improved after the addition of g-C_3_N_4_. The optical energy gaps exhibited compositional dependence on the g-C_3_N_4_ content, changing from 5.23 to 5.34 eV for indirect allowed transitions. Furthermore, the refractive index of the blended films increased from 1.83 to 3.96 as the content of g-C_3_N_4_ changed from 0.0 to 1.0 wt.%. These findings underscored the potential of g-C_3_N_4_ as an effective sensing material, attributed to its advantageous properties, including high surface area, chemical stability, and interaction capabilities with analytes. In summary, this study affirmed the viability of g-C_3_N_4_-enhanced PVC/PVP polymer nanocomposites for practical implementation in optoelectronic fiber sensor applications. Yao et al. [151] summarized recent advances in using fullerene-based materials, such as fullerene/semiconductors and fullerene/non-semiconductors for photocatalytic applications. The report showed that fullerene can be potentially incorporated into semiconductors to enhance their photocatalytic activity for effective wastewater treatment due to its unique optical and photochemical characteristics.

**Figure 15:**
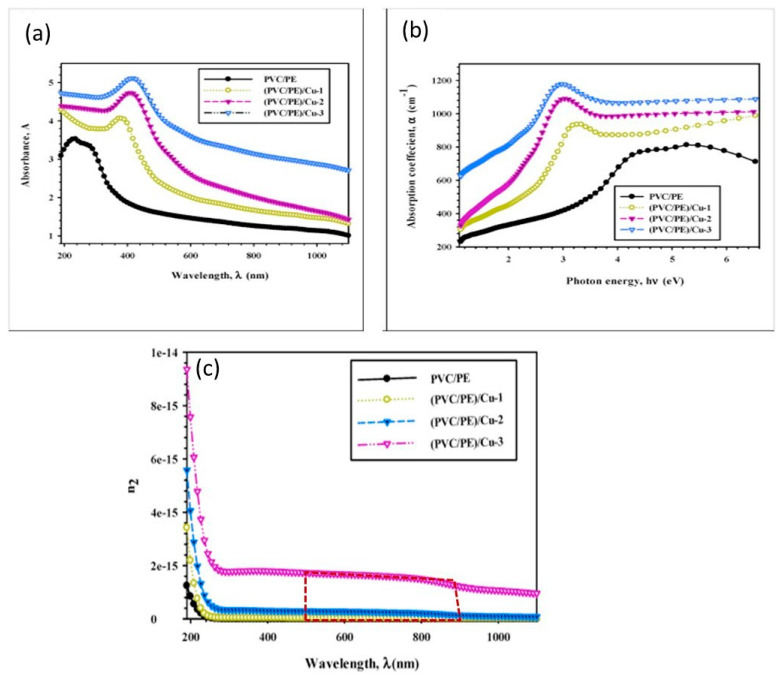
(**a**) Absorbance spectra of PVC/PE for different Cu concentrations, (**b**) absorption coefficient (α) versus the incident photon energy (hν), (**c**) nonlinear refractive index for different Cu % [152].

Elbasiony et al. [152] synthesized PVC/PE polymer nanocomposites with 0–3 wt.% copper (Cu) nanoparticles using a melt extrusion approach for potential applications in photonics. The composites displayed enhanced linear and nonlinear optical susceptibilities with an optical absorption bandgap shifted from 3.38 eV to 2.19 eV as the Cu concentration increased from 0 to 3 wt.% (Figure 15a), indicating enhanced visible light absorption. The nonlinear refractive index varied from 1.2 × 10^−8^ cm^2^/W to 2.1 × 10^−8^ cm^2^/W across the 500–900 nm wavelength range for 0–3 wt.% Cu (Figure 15c).

**Figure 16:**
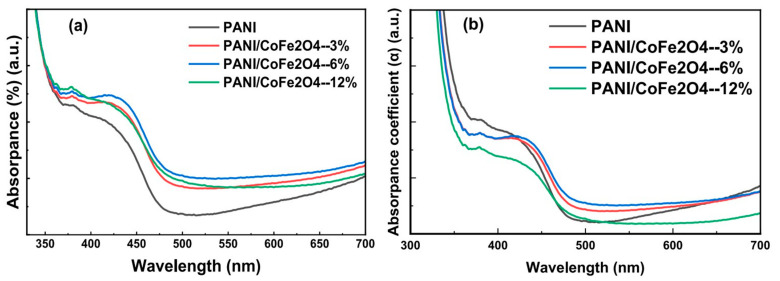
UV–Vis absorption properties of PANI/CoFe_2_O_4_ nanocomposites: (**a**) normalized absorbance and (**b**) absorption coefficient (α) vs wavelength [154].

Karatay et al. [153] explored the nonlinear and saturable absorption behaviors of TiO_2_/carbon-based nanocomposites in Polymethyl Methacrylate (PMMA) using the Z-scan technique. The research found that nonlinear absorption coefficients are higher in the nanosecond regime, while saturation intensity thresholds are higher in the femtosecond regime, attributed to localized defect states. Thermal annealing reduced these defects, leading to lower nonlinear absorption coefficients and saturation intensity thresholds, indicating improved material properties for optical applications. Al-Gharram et al. [154] investigated hybrid Polyaniline/Cobalt ferrite (PANI/CoFe_2_O_4_) nanocomposites synthesized via electrochemical polymerization, focusing on their structural, dielectric, and optical properties. The incorporation of CoFe_2_O_4_ nanoparticles altered the crystallite size, increased the energy band gap, and enhanced the absorbance spectrum, leading to improved optical parameters, as presented in Figure 16. Additionally, the free carrier-to-mass ratio and electrical conductivity increased with higher nanoparticle content, highlighting the potential of these nanocomposites for optoelectronic applications.

## 3. Challenges in Nanocomposite Development

Despite significant advancements in the use of nanomaterials to enhance the performance of composites, some challenges have limited their production and widespread applications. These challenges involve various aspects, ranging from the manufacturing process and production cost to environmental impact. 

### 3.1. Dispersibility

The dispersion of nanoparticles into matrix materials during nanocomposite production is exceedingly challenging. Nanomaterials tend to agglomerate or clump together due to their high surface area, resulting in the non-uniform dispersion of particles within the composite matrix [155,156]. Poor dispersion or non-uniform distribution of nanomaterials can significantly contribute to the formation of voids and porosity within the composite structure. This phenomenon diminishes interfacial bonding and creates weak stress concentration points. The presence of voids or porosity in composites compromises the effectiveness of stress transfer mechanisms between the fiber and polymer matrix, thereby facilitating crack formation and propagation [156,157]. Furthermore, excessive introduction of nanofillers into polymeric resin beyond a certain optimum content may increase the viscosity of the resin. High resin viscosity reduces the volume fraction of the matrix and hinders proper interfacial interaction between the matrix and reinforcement. Weak interfacial interaction leads to a significant reduction in stress transfer and induces crack propagation, ultimately resulting in poor mechanical properties [158]. Therefore, achieving a uniform dispersion of nanomaterials within a polymer matrix is crucial for optimal performance. Proper mixing techniques during composite fabrication have been used to solve this challenge. This involves extended mixing time, employing sonication or high-shear mixing methods, as well as controlling mixing conditions, such as temperature, pressure, humidity, and nanoparticle concentration to enhance proper and uniform dispersion. The use of additives or surfactants to improve the dispersion of nanomaterials in the polymer matrix has also been widely reported as a way of addressing the dispersibility problems, preventing the agglomeration and clumping of nanoparticles.

Ahangaran et al. [159] explored the challenges and solutions associated with metal oxide nanoparticles (NPs), particularly their tendency to aggregate due to high surface energy. The review highlights the importance of chemical surface modification, specifically using silane modifiers, to enhance the dispersion and stability of NPs. The paper covers the synthesis, surface thermodynamics, and salinization kinetics of metal oxide NPs, emphasizing that surface modification effectively prevents agglomeration and improves the stability of NPs across various applications, such as catalysis, drug delivery, and polymer composites.

### 3.2. Compatibility

Nanomaterials often present significant challenges when used in composite materials due to their chemical incompatibility with most polymeric resins. This incompatibility can lead to poor adhesion between the nanomaterials and the matrix, resulting in weak interfacial bonding and suboptimal performance of the composite. Addressing these issues requires a multi-faceted approach that involves several critical processing steps. Firstly, surface treatments of both the nanomaterials and the polymer matrix are often necessary to modify their surface chemistry. These treatments can include functionalization processes that introduce reactive groups onto the surface of the nanomaterials, enabling better chemical bonding with the matrix [109]. For example, introducing hydroxyl or carboxyl groups onto the surface of nanomaterials can improve their wettability and compatibility with polar polymeric resins.

Secondly, the selection of appropriate fabrication techniques is crucial. Techniques such as in situ polymerization, solution blending, or melt blending can be tailored to ensure uniform dispersion of nanomaterials within the matrix, minimizing agglomeration and enhancing the mechanical properties of the composite. The chosen fabrication method must account for the specific interactions between the nanomaterials and the polymer matrix to maximize the efficacy of the composite. Peng et al. [160] conducted a comprehensive review examining the incorporation of functionalized nanofillers into polymers via photopolymerization. The study emphasizes the crucial role of surface modification in enhancing the compatibility of nanofillers with polymers and improving polymerization behavior. It discusses recent advancements in surface modification techniques, their impact on interfacial interactions, and the resulting properties of nanocomposites.

Additionally, the formulation and optimization of the composite require precise control over the concentration and distribution of nanomaterials within the matrix. This involves determining the optimal loading of nanomaterials to achieve the desired balance between strength, flexibility, and other mechanical properties [161,162]. Optimization also includes fine-tuning the processing parameters, such as temperature, pressure, and mixing speed, to ensure consistent quality and performance. Compatibility testing between the nanomaterials and the polymer matrix is another critical step. This involves evaluating the interfacial interactions through various characterization techniques, such as scanning electron microscopy (SEM), transmission electron microscopy (TEM), and spectroscopy. These tests help identify any potential issues with adhesion or dispersion and guide further adjustments to the composite formulation [163]. 

### 3.3. Production Cost

Compared with conventional composite materials, nanomaterial-based composites are more expensive to produce because of the need for intricate processing techniques, specialized equipment, and expert knowledge. These factors contribute to elevated overall manufacturing costs for the final nanocomposite products. In addition, the costs associated with synthesizing materials at the nanoscale, designing and testing various nanomaterials before their utilization, and developing optimal compositions to enhance the properties of the resulting nanocomposite further contribute to the economic challenges of manufacturing. Hence, for certain essential applications, the high costs of nanocomposite production may render them economically non-feasible [164]. Minimizing the production costs of nanocomposites can be addressed by implementing efficient and cost-effective manufacturing processes. This can be achieved by investing in R&D, streamlining production methods, reducing processing steps, maximizing resource utilization, and minimizing material waste through recycling practices. Furthermore, developing efficient manufacturing and recycling processes for waste materials generated during nanocomposite production will not only contribute to cost savings but also significantly minimize the environmental impact.

### 3.4. Environmental Impact

Nanocomposites offer a range of promising properties and applications across various industries. However, like any emerging technology, the environmental impact of nano-based nanocomposites warrants careful consideration, as it depends largely on the different types of nanoparticles used in composite development. Handling and disposal of nano-based nanocomposites can release nanoparticles into the environment, which may have adverse effects on ecosystems and human health if they are not properly managed. The small size of nano-based materials can make them very reactive and potentially hazardous to humans and the environment in general. Nanoparticles can penetrate many biological barriers, such as cell membranes, and may harm cells, tissues, and lungs [165,166,167]. Some nanomaterials used in nanocomposites have shown potential toxicity under certain circumstances. The behavior of nanoparticles in the environment under different conditions has not been documented. Understanding their behavior will help mitigate any adverse effects they may have on ecosystems and human health. In addition, while bioplastics are widely praised for their sustainability and biodegradability, in nanocomposite development, the addition of nanomaterials such as carbon nanotubes or metal nanoparticles can undermine their eco-friendly properties. Many synthetic nanomaterials do not break down naturally, leading to long-term environmental accumulation and posing risks to ecosystems and biodiversity [168]. 

Therefore, it is essential to carefully manage the production and use of nanomaterials in composite development and other applications by conducting comprehensive life cycle assessments (LCAs) to evaluate the environmental impacts associated with nano-based nanocomposites. This involves evaluating the environmental footprint of these materials from raw material extraction to end-of-life disposal. In addition, adequate regulatory frameworks are essential to ensure the safe production, use, and disposal of nanocomposites. Governments and international organizations must develop policies and guidelines to address environmental concerns and minimize potential risks. Finally, the exploration of green-based nanomaterials such as cellulose nanofibrils (CNFs), bacterial cellulose (BC), cellulose nanocrystals (CNCs), chitosan nanoparticles (CnPs), and green-synthesized metal nanoparticles in nanocomposite development is essential. This exploration, coupled with the adoption of more eco-friendly manufacturing processes, will address these environmental and health concerns without significantly compromising their performance and functionality.

## 4. Applications of Nanocomposites

The applications of nanocomposite materials are vast due to their unique and numerous attractive characteristics, as well as their ability to perform excellently in many critical applications compared with other traditional engineering materials. Some potential applications of nanocomposites include energy storage, wastewater treatment, food processing, and the biomedical sector [169,170,171]. Recent advances in nanocomposite technology in the biomedical sector have effectively addressed the challenges posed by highly immunosuppressive tumors, by inducing robust immunotherapeutic responses through synergistic functions introduced by various nanosized molecules. The ability to tailor nano-based composite materials to specific properties, such as biocompatibility and controlled release of drugs, encourages their widespread application in the biomedical sector for drug delivery, medical implants, disease diagnosis, and bioimaging [172,173]. Nanocomposites have also found interesting applications in aerospace and automobile parts production. These multiscale composites generally exhibit a high strength-to-weight ratio, making them attractive materials in automotive and aerospace applications for enhancing fuel efficiency, reducing emissions, and ensuring long-term durability [174,175,176]. They are used in the design of specific parts such as general body panels, engine components, and tires. In the energy sector, nanocomposites have been widely used for energy storage and conversion. They are used in components such as supercapacitors, solar cells, fuel cells, and batteries with improved performance and reduced size [177]. Nanocomposites play an increasingly important role in the advancement of lithium-ion batteries (LIBs). By incorporating nanoscale materials into the electrodes, nanocomposites enhance LIB performance by improving energy density, cycling stability, and charge-discharge rates. The large surface area and unique properties of nanomaterials, such as carbon nanotubes, graphene, and metal oxides, enable better electron transport and lithium-ion diffusion, leading to batteries with higher capacity, longer life, and faster charging capabilities. These advancements have been documented in several studies.

Hong et al. [178] demonstrated the potential of MXene-TiO_2_ nanocomposites for lithium-ion battery applications. This study introduces a method of using a flashlight to heat MXene films, creating a porous MXene-TiO_2_ freestanding anode with enhanced interlayer spacing and TiO_2_ formation. The resulting anode shows nearly 50 times higher lithium-ion storage capacity (148 mAh/g) and excellent cycle stability over 1500 cycles without degradation. Yu et al. [179] developed a cost-effective, eco-friendly method to synthesize carbon-supported lithium iron phosphate (LFP/C) cathodes using a metal-organic framework precursor. The LFP/C composite shows enhanced lithium-ion storage with a reversible capacity of 109.7 mAh/g after 500 cycles and good low-temperature performance. The synthesis process is straightforward and scalable, promising improved rate capability and cycle stability for lithium-ion batteries. Khan et al. [180] summarized the potential of graphene and its composites as promising next-generation anodes due to their enhanced conductivity and reduced volume expansion. The study highlighted the limitations of commonly used graphite anodes in lithium-ion batteries (LIBs) due to their limited capacity and demonstrated how graphene-based nanocomposites could serve as potential alternatives for future LIB anodes.

**Figure 17:**
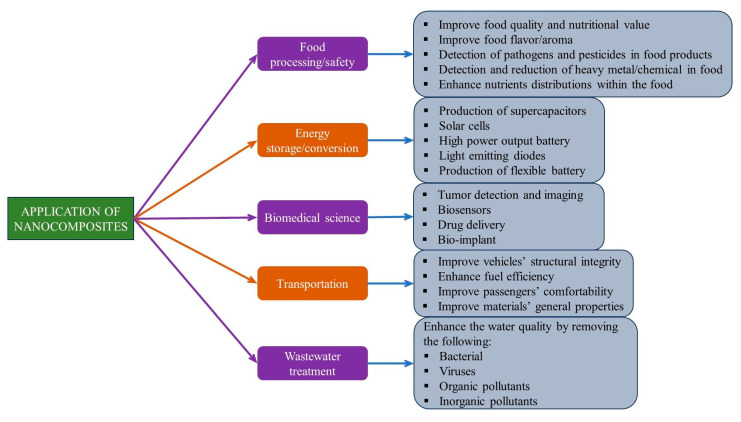
Key application areas of nanocomposite materials.

Organic-based nanocomposites, such as nanocellulose-modified polymer composites, have been widely used in wastewater treatment because of their significant adsorption capacity, photocatalytic degradation, anti-biofouling effect, nanofiltration efficiency, and environmental friendliness [181]. Namasivayam et al. [182] demonstrated the effectiveness of nanocellulose acetate membrane technology in wastewater treatment. Their findings highlight a sustainable approach to addressing hazardous invasive aquatic weeds by integrating them into the composite membrane, thus leveraging green nanotechnology principles to enhance wastewater treatment efficacy. You et al. [183] investigated the potential of employing environmentally friendly and naturally abundant biomass materials to remove heavy metal ions from wastewater before discharge into the environment. The researchers developed a ternary composite comprising nanocellulose/carbon dots/magnesium hydroxide (CCMg) using a simple hydrothermal method. The heavy ion (Cd^2+^ and Cu^2+^) removal mechanisms by the composite were examined using a combination of experimental techniques and density functional theory calculations. This study demonstrated the efficacy of CCMg in removing heavy metal ions from wastewater, which is attributed to its high adsorption capacity and straightforward recovery operation. The use of nanocomposite materials has witnessed a steady rise across various industries. This growing trend underscores the significance of nanocomposites in addressing diverse industrial needs. Figure 17 highlights the key application areas of nanocomposite materials along with their specific advantages. Following thorough experimental studies, these materials showcase versatile characteristics tailored to meet specific application requirements. Table 2 provides a detailed overview of the use of nanocomposite materials and their reported performance across different sectors.

## 5. Future Outlook

Nanomaterials have already demonstrated great potential in enhancing the performance of nanocomposites in terms of mechanical strength, thermal and electrical conductivity, sensing capability, and barrier properties, paving the way for their wider applications across many industries. Today, researchers are further exploring nanomaterials with captivating and enhanced properties compared with existing traditional nanomaterials. Therefore, the use of nanomaterials in nanocomposite development is expected to grow significantly in the future due to the increasing research interest in this field. Efforts are being directed toward improving the compatibility and dispersion efficiency of nanomaterials within composite matrices, which are crucial for achieving the desired composite properties. This involves optimizing nanomaterial properties and their integration into matrices to achieve superior performance in specific applications, resulting in multifunctional nanocomposites suitable for tasks such as self-healing, shape memory, biomedical implants, and conductive materials for flexible electronics. To address environmental concerns, future developments will focus on environmentally friendly nanocomposite materials that use organic-based nanomaterials as reinforcement phases. This will reduce reliance on fossil-based resources, minimize waste generation, and mitigate environmental and health risks. Renewable nanomaterials sourced from cellulose, chitin, or lignin, along with eco-friendly synthesis methods and recycling processes, will play a crucial role in this endeavor. Nanocomposites exhibit remarkable potential in advanced and specialized fields. As the technology of merging nanomaterials into composite matrices continues to progress, there is a likelihood of witnessing more economical and efficient manufacturing techniques in this sector, which will further widen the application of nanocomposites across diverse industries. At present, nanocomposites are used in various applications; however, as research in this area advances, there are several potential future applications of nano-based materials and nanocomposites in critical and advanced sectors of the economy such as quantum computing, energy harvesting and storage, tissue engineering, food packaging and safety, as well as nanomedicine. 

In summary, the future holds immense potential for nanomaterials and nanocomposites, driving innovation and addressing complex challenges across critical and advanced sectors of the economy. Continued research and development efforts will unlock new possibilities and create advanced nanocomposite materials through nanotechnology.

## 6. Conclusions

Nanomaterials are valuable additives for enhancing the overall properties of composite materials owing to their unique characteristics. The incorporation of nanomaterials into polymer matrices has led to the development of polymer-based nanocomposites featuring superior mechanical strength, enhanced electrical conductivity, improved thermal stability, and other critical attributes. These advancements open up new horizons for applications in diverse fields such as transportation, construction, sports, electronics, and biomedicine. However, the widespread adoption of nano-based materials in composite development faces certain limitations that have not been thoroughly delineated. These constraints encompass the high cost associated with nanomaterials and their synthesis, elevated manufacturing expenses related to the production of nanocomposites, and concerns about health and environmental implications linked to nano-based products. To fully unlock the potential benefits of these innovative materials for advanced applications, it is imperative to engage in sustained research endeavors within this domain. Addressing challenges such as cost-effectiveness, efficient synthesis methods, and, most importantly, ensuring health and environmental safety will facilitate their comprehensive utilization in composite developments. Continued research efforts in this field are crucial to fully explore the potential benefits of nanocomposites and establish them as essential components for a diverse range of state-of-the-art applications.

## Figures and Tables

**Table 1:** TM concentrations and name conventions of the laminates used in the study [124].

Sample	Name Convention	Concentration of TM Particles (wt.%)
Basalt-fiber reinforced plastic (composite)	BFRP	0
Basalt/epoxy composite with 0.5 wt.% TM (no surfactant)	C1	0.5
Basalt/epoxy composite with 1.0 wt.% TM (no surfactant)	C2	1.0
Basalt/epoxy composite with 2.0 wt.% TM (no surfactant)	C3	2.0
Basalt/epoxy composite with 1.0 wt.% TM (with surfactant)	C4	1.0 + surfactant

**Table 2:** Summary of nanocomposite development and utilization across various sectors.

Nanocomposites	Area of Application	Performance Evaluation	References
Nanofiltration membranes (NF) made of a thin layer of graphene oxide (GO) and Zn-based metal-organic framework (ZIF-7) nanocomposites deposited on chitosan (CHI)-coated polyethersulfone (PES)	Wastewater treatment	The modified nanocomposite membrane with GO-ZIF-7 demonstrates a superior dye removal efficiency of approximately 94%, surpassing the original CHI-coated membrane, which achieved around 84% removal.	[184]
Pure low-density polyethylene and titanium dioxide nanoparticles (P-LDPE/TiO_2_-NPs)	Food packaging	The nanocomposite films exhibited significant improvements in mechanical properties, antibacterial activity, and permeability measurements. These enhancements demonstrate their promising potential for application in perishable food packaging.	[185]
Graphene/chitosan/polyvinyl alcohol (G/CHI–PVA)	Wound healing	The study demonstrated graphene’s potential as an antibacterial material, which proves beneficial for expediting wound healing. This is achieved by hindering the multiplication of prokaryotic organisms, as observed in the body systems of mice and rabbits.	[186]
Carbon nanotubes (CNTs) functionalized with polyaniline (PANI-CNTs), grafted on CoNi(PO_4_)_2_ (PANI-CNTs/CoNi(PO_4_)_2_	Asymmetric supercapacitor	The hierarchical structured CoNi(PO4)2 with a mass of 40 mg of PANI-CNTs (CNP40) exhibited an enhanced specific capacity of 1268 Cg^−1^ (2136 F g^−1^ at 1.5 F g^−1^) while demonstrating excellent diffusive behavior (b = 0.5).	[187]
Molecularly imprinted polymers (MIPs) doped with SWCNT and POSS	Drug delivery	MIPs containing POSS and SWCNT showed superior controlled release in vitro. In vivo studies revealed that POSS-SWCNT MIP reached maximum plasma concentration after 4 h, exhibiting a significantly higher AUC0-9 (544.73 ng h mL^−1^) compared to control MIPs and NIP (327.48, 212.91, 230.35, and 275.13 ng h mL^−1^ for POSS MIP, SWCNT MIP, MIP, and POSS-SWCNT NIP, respectively).	[188]
MWCNT functionalized PAMAM dendrimer (d-MWCNT) reinforced with strontium-substituted hydroxyapatite HAP (SrHAP) (d-MWCNT-SrHAP)	Bone tissue engineering	The composite demonstrated a significant increase in curcumin loading and delayed release compared to SrHAP, and in vitro studies with human osteoblast-like cells indicated enhanced osteoblast activities. The findings indicate that d-MWCNT-SrHAP-C holds promise as an effective drug delivery system for applications in hard tissue engineering.	[189]
Carbon fibers (CFs)/multiwalled carbon nanotubes (MWCNTs)	Artificial wearable electronics	The hierarchical composites exhibit outstanding attributes, including enhanced high-pressure sensing (42.7 kPa), rapid response (relaxation times < 100 ms), a broad working range (0–60 kPa), and excellent stability over 6000 cycles. Additionally, the composites exhibit high thermal sensitivity (2.46 °C^−1^ between 30 and 40 °C), highlighting their potential in multifunctional wearable electronics.	[190]
Biphasic polyolefin system, consisting of macro-polyethylene (PE) phase, micro polypropylene (PP) phase, and nanostructured graphene (PE/PP/G)	Lightweight transportation industry	The newly developed nanocomposite exhibits remarkable thermo-mechanical properties, making it a potential lightweight thermoplastic olefinic nanocomposite suitable for the transportation sector.	[191]
Polypropylene (PP)/graphene composites (PP/G)	Electronic and thermal management	The composite material achieved a significantly high through-plane thermal conductivity of 10.93 W·m^−1^·K^−1^, approximately 55 times greater than pure PP. It demonstrates exceptional heat dissipation properties, making it suitable for use in LED integration for efficient thermal management.	[192]
Nanoclay, montmorillonite (MMT)/chitosan (Cs) biopolymer matrix	Bioelectricity generation and wastewater treatment in microbial fuel cells	The addition of MMT in the Cs matrix demonstrated enhanced performance in terms of power generation and chemical oxygen demand (COD) removal. The results showed that biopolymer Cs-based nanocomposite incorporated with MMT can be effectively used as an alternative for bio-proton exchange membrane in microbial fuel cell applications.	[193]
RGO-based SiO_2_-TiO_2_ nanocomposite	Supercapacitor for energy storage devices	The RGO-SiO_2_-TiO_2_ supercapacitor exhibited a maximum energy density of 35 Wh/kg at 630 W/kg power density, maintaining 84% capacitance stability after 10,000 cycles. This approach offers a cost-effective solution for mitigating CO_2_ emissions and creating efficient energy-harvesting devices.	[194]
Carbon nanotubes/nickel sulfide/cobalt sulfide (CNTs/NiS/CoS) nanocomposites	Supercapacitor applications	The CNTs/NiS/CoS nanocomposite displayed significantly lower impedance compared with CNTs alone. In a 3 M KOH solution, it exhibited a specific capacitance of 1249.88 mAh/g at 1 A/g, retaining 97.17% after 8000 cycles. Notably, the composite demonstrated impressive energy density (624.44 Wh/kg) and power density (8325.87 W/kg), emphasizing its potential for high-performance supercapacitor applications.	[195]

## Data Availability

No data were used for the research described in this article.
